# Expression of fatty acid related gene promotes astaxanthin heterologous production in *Chlamydomonas reinhardtii*

**DOI:** 10.3389/fnut.2023.1130065

**Published:** 2023-03-20

**Authors:** Jin-peng Sun, Xue-hong Wei, Xiao-mei Cong, Wen-hua Zhang, Le-Xin Qiu, Xiao-nan Zang

**Affiliations:** Key Laboratory of Marine Genetics and Breeding, Ocean University of China, Ministry of Education, Qingdao, China

**Keywords:** *Haematococcus pluvialis*, *Chlamydomonas reinhardtii*, astaxanthin, fatty acid, gene heterologous expression, metabolic engineering

## Abstract

Natural astaxanthin is a high-value ketone carotenoid mainly derived from *Haematococcus pluvialis*, which is an excellent antioxidant for human consumption. To study the role of lipids in accumulation of astaxanthin, the *H. pluvialis*-derived astaxanthin synthesis pathway genes (β-carotene ketolase gene, *BKT* and β-carotene hydroxylase gene, *BCH*) and fatty acid elongation gene (mitochondrial trans-2-enoyl-coa reductase gene, *MECR*) were heterologously co-expressed in *C. reinhardtii*. Zeaxanthin, the precursor of astaxanthin synthesis, was significantly increased after *BKT* and *BCH* were expressed. In contrast, the α-carotene that competes with astaxanthin synthesis for lycopene decreased significantly. This redistribution of carbon flow was conducive to the synthesis of astaxanthin. In addition, the transformant only expressed astaxanthin metabolism related genes (*BKT*, *BCH*) would lead to an increase in total lipid, a decrease in monounsaturated fatty acids and an increase in polyunsaturated fatty acids. On this basis, the expression of *MECR* gene further increased the total lipid, and the relative content of different fatty acids also changed. The astaxanthin content of algal strains transformed with *BKT* and *BCH* genes was nearly 50% higher than that of the wild type. On this basis, the astaxanthin content of transformants expressing *MECR* gene related to long-chain fatty acid synthesis was increased by 227.5%. In this study, an astaxanthin production model similar to *H. pluvialis* by combining carotenoid metabolism and fatty acid metabolism was constructed in *C. reinhardtii*. The results suggest that the increase in astaxanthin is indeed linked to the regulation of fatty acid metabolism, and this link may involve the type of fatty acids and the dynamics of astaxanthin ester in cells. The strategy of promoting the synthesis of fatty acids has potential to achieve efficient production of astaxanthin in *C. reinhardtii*.

## 1. Introduction

Since it was discovered in 1844, *Haematococcus pluvialis* has attracted extensive attention due to its unique astaxanthin productivity (1, 2). At present, *H. pluvialis* is cultured for large-scale production of natural astaxanthin, which is widely used in nutritional supplements, cosmetics and aquaculture feed because of its conspicuous antioxidant activity (3). “Two-stage culture method” of *H. pluvialis* is adopted in the industrial production of natural astaxanthin. In this model, the accumulation of biomass and the synthesis of astaxanthin are separated in different time stage. Two-stage method can achieve large-scale production, but requires a long incubation time and is limited by specific conditions to induce astaxanthin. In addition, stress induction will lead to the decline of biomass (4).

As model alga, *Chlamydomonas reinhardtii* is closely related to *H. pluvialis*, and has mature genetic operation method, clear genetic background and superior growth rate than *H. pluvialis* (5). The cell doubling time of *C. reinhardtii* is 5-6 h, which is much shorter than that of *H. pluvialis* (>25 h) (6). In addition, compared with the thick cell wall of *H. pluvialis*, some kinds of *C. reinhardtii* with cell wall defect can significantly reduce the cost of astaxanthin extraction (7). What’s more, compared with bacteria and fungi, *C. reinhardtii* has a major advantage as a host organism for astaxanthin production because it can grow heterotrophically and use CO_2_ in the air without requiring other organic carbon, which is of great significance for energy conservation and carbon emission reduction.

In *H. pluvialis*, β-carotene is catalyzed to produce astaxanthin by β-carotene ketolase (*BKT*) and β-carotene hydroxylase (*BCH*). In *C. reinhardtii*, β-carotene is catalyzed by hydroxylase to produce zeaxanthin, which then circulates through xanthophyll cycle to produce antheraxanthin and violaxanthin but without synthesizing astaxanthin (8–12). β-carotene ketolase (*BKT*) and β-carotene hydroxylase (*BCH*) are the two key enzymes in the astaxanthin synthesis of *H. pluvialis*. β-carotene is the substrate of these two enzymes to produce astaxanthin, but there are many intermediate products in the reaction process and the reaction efficiency is low. β-carotene reacts with *BCH* to produce zeaxanthin, which is then ketogenic to astaxanthin. If β-carotene reacts with *BKT* first, the intermediate products are echinenone and canthaxanthin, and astaxanthin will be further produced under the catalysis of *BCH* (13). Both *C. reinhardtii* and *H. pluvialis* are unicellular green algae, the synthesis of carotenoid in *C. reinhardtii* is similar to the synthesis of astaxanthin precursor substances in *H. pluvialis*. *C. reinhardtii* also produces β-carotene, but the synthesis of astaxanthin is limited. Therefore, using *C. reinhardtii* expression system to study the function of astaxanthin synthase has an incomparable advantage.

In *H. pluvialis*, the accumulation of astaxanthin is related to the synthesis of glyceride, and a large number of astaxanthin combines with unsaturated fatty acids in the form of astaxanthin monoester (94%). The related fatty acids are mainly C18 family unsaturated fatty acids (C18:1, C18:2 and C18:3) (14, 15). Previous research negates the correlation between astaxanthin and fatty acids at the transcriptional level, but there is feedback regulation at the metabolic level. The astaxanthin consumed by esterification can promote the synthesis of astaxanthin (16). In addition, the accumulation and diffusion of astaxanthin esters in cells depend on the triacylglycerol existing in cyclonic lipid bodies. Intriguingly, the synthesis of astaxanthin did not seem to be related to the content of triacylglycerol in cells (17, 18). Therefore, the relationship between astaxanthin synthesis and fatty acid accumulation needs further study, which will provide guidance for improving the level of astaxanthin.

Previous studies have shown that the genes for astaxanthin synthesis in *C. reinhardtii* is not fully expressed (19). The content of astaxanthin in wild type *C. reinhardtii* is very low and even undetectable. Therefore, firstly, *BCH* and *BKT* genes deriving from *H. pluvialis* were expressed into *C. reinhardtii* to catalyze β-carotene to build a production pathway of astaxanthin. In *C. reinhardtii* and *H. pluvialis*, the precursor substances of carotenoids, such as isopentenyl pyrophosphate (IPP), comes from methyl ethyl phosphate (MEP) pathway (20). In alga cells, MEP pathway competes with *de novo* synthesis of fatty acids for acetyl-CoA. Moreover, previous studies have shown that astaxanthin is mainly esterified with long-chain fatty acids. Therefore, in order to avoid the decrease of astaxanthin accumulation caused by excessive carbon precursors flowing to fatty acid synthesis, we did not aim at the *de novo* synthesis of fatty acids but to selected genes related to fatty acid carbon chain elongation. In previous studies in our laboratory, it was found that mitochondrial trans-2-enoyl-coa reductase gene (*MECR*) relating the elongation of fatty acids in mitochondria was up-regulated when *H. pluvialis* was induced to accumulate astaxanthin under high light. So *MECR* derived from *H. pluvialis* was cloned to express in *C. reinhardtii* to improve the lipid metabolism. It is hoped that the yield of astaxanthin in *C. reinhardtii* can be further increased, and the analysis basis and reference for the interaction between astaxanthin and fatty acids can be provided.

## 2. Methods and materials

### 2.1. Culture of algae

*H. pluvialis* OUC H2 used in this experiment was obtained from the Algae Genetics and Breeding Laboratory of Ocean University of China. Green growth stage: Bold Basal Medium was used. Light intensity was set as 30 μmol·m^−2^·s^−1^ (light/dark, 12 h/12 h), the temperature was 23 ± 1°C. Red induction stage: BBM was replaced by oligotrophic medium containing NaAc and Fe^2+^, the light intensity was 195 μmol·m^−2^·s^−1^ (light/dark, 12 h/12 h) and temperature was 23 ± 1°C. *C. reinhardtii* CC849 was obtained from Chlamydomonas Resource Center, University of Minnesota, United States. Alga was cultured by TAP (Tris Acetate Phosphate) medium. The light intensity was 50 μmol·m^−2^·s^−1^ (light/dark, 12 h/12 h), the temperature was 23 ± 1°C, 100 rpm.

### 2.2. Extraction of DNA and RNA

Total DNA of *H. pluvialis* OUC H2 was extracted by using Takara MiniBest Plant Genomic DNA Extraction Kit after grinding with liquid nitrogen. *C. reinhardtii* was shaken with glass beads at 3000 rpm for 5 min to break cells, and then DNA was extracted according to the instructions of the same kit. E.Z.N.A plant RNA Kit (Omega) was used to extract RNA, and then reverse transcription was performed by using 5 × PrimeScript RT Master Mix (Perfect Real Time, Takara) to construct cDNA library. Gel purification was carried out with Gel Extraction Kit (200) (Omega). Plasmid Miniprep Purification Kit (GMbiolab) was used to extract plasmids from *Escherichia coli*.

### 2.3. Genes cloning and construction of the transformation vectors

The primers for cloning *BKT*, *BCH*, *MECR* are displayed in Table S1. The cDNA and DNA of *H. pluvialis* OUC H2 was used as template. TA cloning was performed to construct cloning vector (Tsingke Biotechnology, China) for sequencing (Sangon Biotech, China). The plasmid pUC19 was used as a transition vector to add promoter and terminator, and then the expression frame was linked into the *C. reinhardtii* expression vector pHyg3. All three genes use β-2 tubulin promoter (P_β2-Tub_) and rbcS2 terminator (T_rbcS2_) from pHyg3. The sequences were constructed into the vector by enzyme digestion and ligation. The restriction enzymes used are listed in the Table S1. The expression vectors carrying different genes are, respectively, named as pHyg3-*BKT*-*BCH* and pHyg3-*BKT*-*BCH*-*MECR*.

### 2.4. Transformation of *Chlamydomonas reinhardtii*

The alga was centrifuged (4,000 g, 10 min) and suspended by fresh TAP medium to prepare cell suspension with cell density of 10^8^ cells/ml. 400 μl algal cells were mixed with 5–10 μg plasmid DNA and 400 mg sterile glass bead, and then vortexed for 25–45 s. Then fresh TAP medium was inoculated and the algal cells were cultured under dark environment of 23°C and 50 rpm agitation for 18 h. After centrifuged the recovered alga (4,000 g, 10 min) and added 400 μl fresh TAP medium, 100 μl of culture was spread on the resistant plate (with 10 μg/ml hygromycin B) (21). Positive algal colonies were observed at about 15 days later. Because the glass bead-based transformation of a foreign vector carrying the foreign gene(s) is generally randomly inserted in the genome, at least 20 clones were selected for further research. The algae strains with good growth state, stable gene integration and high abundance of expressed products were used for functional studies.

### 2.5. Southern blot analysis

Two groups of different endonucleases were used to digest genomic DNA (37°C, 3 h). The information of genes and enzymes is listed in Table S2. The digestion products were separated by agarose gel electrophoresis, then denatured solution (1.5 mol/l NaCl，0.5 mol/l NaOH) was added to break the double strand. And then, the denatured nucleic acid was transferred to nylon membrane (0.22 μm, Pall, United States) for fixation after washing with neutralization solution (0.5 mol/l Tris–HCl，1.5 mol/l NaCl). Then the digoxigenin labeled probe was prepared by Random Priming method and hybridized with genomic DNA. The primers used to prepare the probe are listed in Table S3.

### 2.6. Quantitative real-time PCR

Transformants and wild type were cultured at normal or high light intensity for 9 days, and the RNA was extracted every 3 days. The primers used for amplification of exogenous *BCH*, *BKT*, *MECR* were designed by the gene sequences from *H. pluvialis* (Table S4). PCR products were then quantified continuously with the BIOER LineGene 9,640 using TB Green Premix Ex Taq II (Takara) according to manufacturer’s instructions. After heating at 95°C for 30 s, cycling parameters were: 40 cycles of 95°C for 5 s, 56°C for 20 s. Finally, the specificity of the qRT-PCR products was confirmed by performing a melting temperature analysis at temperatures ranging from 60°C to 95°C at 4°C/s. Transcription levels of the target genes were calculated by the 2^−ΔΔCT^ method. To standardize the results, the relative abundance of *RCK* gene (G protein beta subunit) was also determined and used as the internal standard.

### 2.7. Determination of *Chlamydomonas reinhardtii* biomass

Cell density: The absorbance of the algal solution was measured to calculate the cell density according to the standard curve obtained in the previous: *y* = OD_750_ × 1.2335 × 10^7^ (cells/ml). Dry cell weight: The algal liquid was centrifuged for 10 min at 4000 rpm. The algal cell precipitation was washed with ddH_2_O twice. The clean algal mud was dried in a freeze-dryer (Shanghai Bilang Instrument Manufacturing) for 6–7 h until the algal mud was completely dry and powdered. Then, the weight of algal powder was measured.

### 2.8. Pigments extraction and analysis

Total carotenoids from *C. reinhardtii* were extracted by using 80% of acetone as previously described (22). Acetone was added to the algal powder. After ultrasonic crushing, 0.22 μm filter membrane was used for filtration. Then the samples were analyzed by HPLC using Thermo UItiMate 3,000 UHPLC chromatograph (Thermo, United States). The analytical conditions: YMC Carotenoid S-5 μm column (4.6 × 250 mm, 5 μm), flow rate = 1 ml/min, detection, 450 nm. Mobile phase: A: MeOH: MTBE: H_2_O (81:15:4); B: MeOH: MTBE (6.5:93.5). The sample size was 5 μl. The determination of carotenoids was achieved by the comparison of characteristic absorption spectrum and reduced retention time with standards. And they were quantitatively determined by external standard method. 100 ml methanol was used to dissolve lutein (20 mg), zeaxanthin (5 mg), β- Cryptoxanthin (40 mg), α- Carotene (5 mg), β- Carotene (20 mg), neoxanthin (10 mg), violaxanthin (10 mg). The standard solution was mixed and use 0.1% BHT-acetonitrile: methanol: dichloromethane (75,20:5) to prepare 1/100, 5/100, 10/100, 20/100, 40/100, 60/100, 80/100 μg/ml of working fluid. Astaxanthin standard was prepared with concentration gradient of 0.1, 0.2, 0.5, 1, 2, 5 μg/ml. Carotenoid content of sample (μg/g) = (c × V × F) / m, where c is the concentration of carotenoids in the sample calculated according to the standard curve, V is the volume of the extract, m is the sample weight of about 0.2 g, and F is the dilution factor.

To determine the astaxanthin content in cells, 5 mg of algal powder was resuspended with 5 ml KOH/methanol solution, and then heated at 60°C for 5 min to destroy chlorophyll and convert astaxanthin ester into free astaxanthin. After heating, the algal cells were centrifugated at 4000 rpm for 10 min. After discarding the supernatant, 2 ml DMSO was add to the precipitation for extracting astaxanthin, and the extraction was repeated until the precipitation turns white. The supernatant was combined and diluted to a certain volume with acetone. The absorbance was measured at 474 nm. Finally, the astaxanthin content was calculated according to the formula P = AV/ (10b ε m). In the formula, A is the absorbance at 474 nm of the sample to be measured. V is the constant volume (ml). b is the length of the optical path of the colorimetric cell, 1 cm. ε is the light absorption coefficient of astaxanthin, 0.191 cm^2^/μg. m is the mass of algal powder (mg).

### 2.9. Lipid extraction and determination of fatty acids by GC–MS

3 ml chloroform methanol (v/v, 2/1) mixture was added into 20 mg of algae powder, and fully mixed. Ultrasonic crushing for 15 min, centrifugation of 10,000 g for 10 min, the supernatant was transfer to another centrifuge tube, and repeated twice. 3 ml of tri distilled water was added into the supernatant, and centrifuged 10,000 g for 10 min for stratification. The lower layer of liquid was taken into a glass bottle that has been weighed (m1), and placed in the fume hood until the chloroform volatilizes completely to constant weight. The total mass of the glass bottle and the algae lipid (m2) was weighted, and the total fat content ω was calculated. The calculation formula is as follows: *ω* = (m2-m1)/20 × 100%. Fatty Acid Methylation: The lipid was saponified with KOH-methanol solution to obtain fatty acid. Under the catalysis of BF_3_, fatty acids and methanol formed methyl esters, which are extracted with n-hexane for gas chromatography–mass spectrometry analysis (MassHunter GC/MS, Agilent). The full scan method of GC–MS was used for detection, the NIST standard mass spectrometry library ratio method was used for qualitative analysis, and the peak area percentage method was used for relative percentage content quantification. Gas chromatographic conditions: chromatographic column, DB-INNOWax quartz capillary column (30 m × 0.25 mm × 0.25 μm). The initial temperature of the chromatographic column is 50°C, which shall be kept for 1 min, and the temperature shall be raised to 250°C at 10°C/min, which shall be kept for 5 min. The sample inlet temperature is 250°C. The carrier gas is high-purity helium, in constant flow mode, with a flow rate of 1.0 ml/min. Injection volume is 1 μl. Mass spectrometry analysis conditions: ion source EI. Ion source energy 70 eV. The ion source temperature is 250°C. The scanning mode is full scanning. The mass scanning range is 50 ~ 500 amu. The solvent is delayed for 3 min.

### 2.10. Statistical analyzes

SPSS and Excel software were used for data analysis. The data were obtained as the mean value ± SD. Significant differences were statistically analyzed by ANOVA. Statistical significance was defined as value of *p* <0.05. All experiments were performed at least three biological replicates to ensure reproducibility.

## 3. Results

### 3.1. Construction of a *Chlamydomonas reinhardtii* platform for astaxanthin production

**Figure 1:**
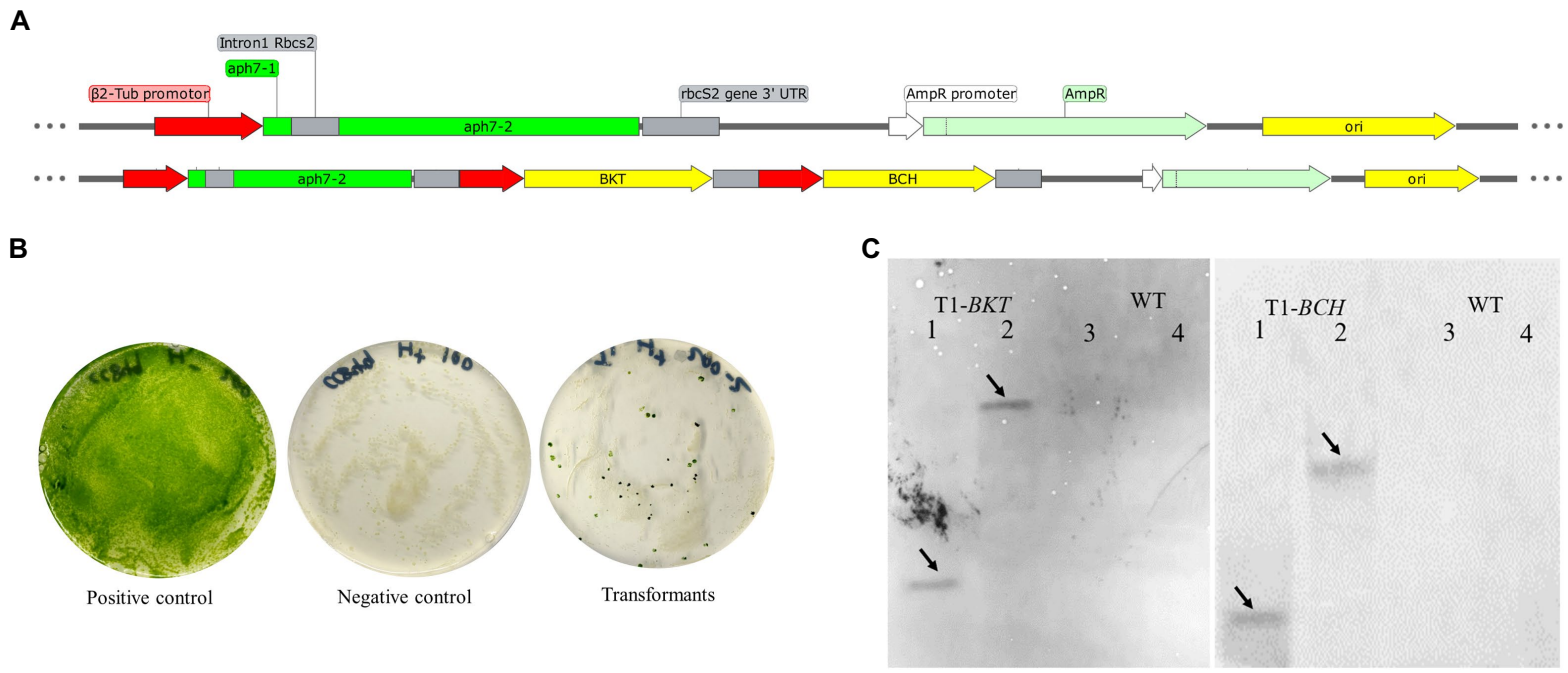
Diagrammatic sketch map of foreign gene expression vector and transformation results of foreign genes. **(A)** The foreign gene expression vector was constructed based on plasmid pHyg3. The names of each sequence are marked in the figure. **(B)** Growth results of transgenic algae strains. The positive control treatment was that the wild type was cultured on the medium without hygromycin B, the algal colony grew normally, and the culture plate was green. The negative control treatment was that the wild type was cultured on the culture medium containing hygromycin B, and the algal colony was completely albino and died 2 weeks later. The transformed algal strain was endowed with hygromycin B resistance and formed single algal colonies on the plate. **(C)** Southern blotting results of *BKT* and *BCH* in T1. The solid line arrows indicate the position of the band. Lane 1, 2 are T1 and 3, 4 are WT. For the detection of *BKT*, 1 and 3 were digested with *Sac*I/*Hin*dIII. Two and Four were digested with *Bam*HI/*Sal*I. For the detection of *BCH*, 1 and 3 were digested with *Bgl*II/*Mlu*I. Two and Four were digested with *Nde*I/*Xho*I.

The structure of expression vector is shown in Figure 1A. The vector pHyg3 was containing the *C. reinhardtii* β-2 tubulin promoter (P_β2-Tub_), rbcS2 gene intron 1, streptomyces hygroscopicus aminoglycoside phosphotransferase gene (*aph7*) and rbcS2 3′ untranslated region (T_rbcS2_). This cassette conferred a resistance against hygromycin B. The P_β2-Tub_ and T_rbcS2_ were cloned to construct a cassette, then *BKT* and *BCH* were introduced into the cassette, respectively. Then *BKT*-cassette and *BCH*-cassette were inserted into vector pHyg3 using the enzyme sites as shown in Table S1 to get the vector pHyg3-*BKT*-*BCH*. All exogenous genes are regulated by *C. reinhardtii* β-2 tubulin promoter and *rbcS2* terminator. The cells grown on the selection medium containing hygromycin (10 μg/ml) were termed as transformants. In order to confirm the stable inheritance of the transformed genes, the transformants were continuously cultured for 10 generations under selective conditions (Figure 1B).

The finally obtained transformant was named as T1 and the untransformed strain CC849 was regarded as WT. Genomic DNA of T1 and WT was extracted for southern blotting analysis (Figure 1C). After DNA was digested with *Sac*I/*Hin*dIII and *Sal*I/*Bam*HI enzymes, it was hybridized with the probe of *BKT* gene. A clear single band appeared in T1. After DNA was digested by *Mlu*I/*Bgl*II and *Xho*I/*Nde*I enzymes respectively, clear bands also appeared in the hybridization with the probe of *BCH* gene. The untransformed algal strains were digested by same enzymes and hybridized with same probes, but no bands appeared, indicating that the transformed *BKT* and *BCH* were integrated into the genomic DNA of T1.

**Figure 2:**
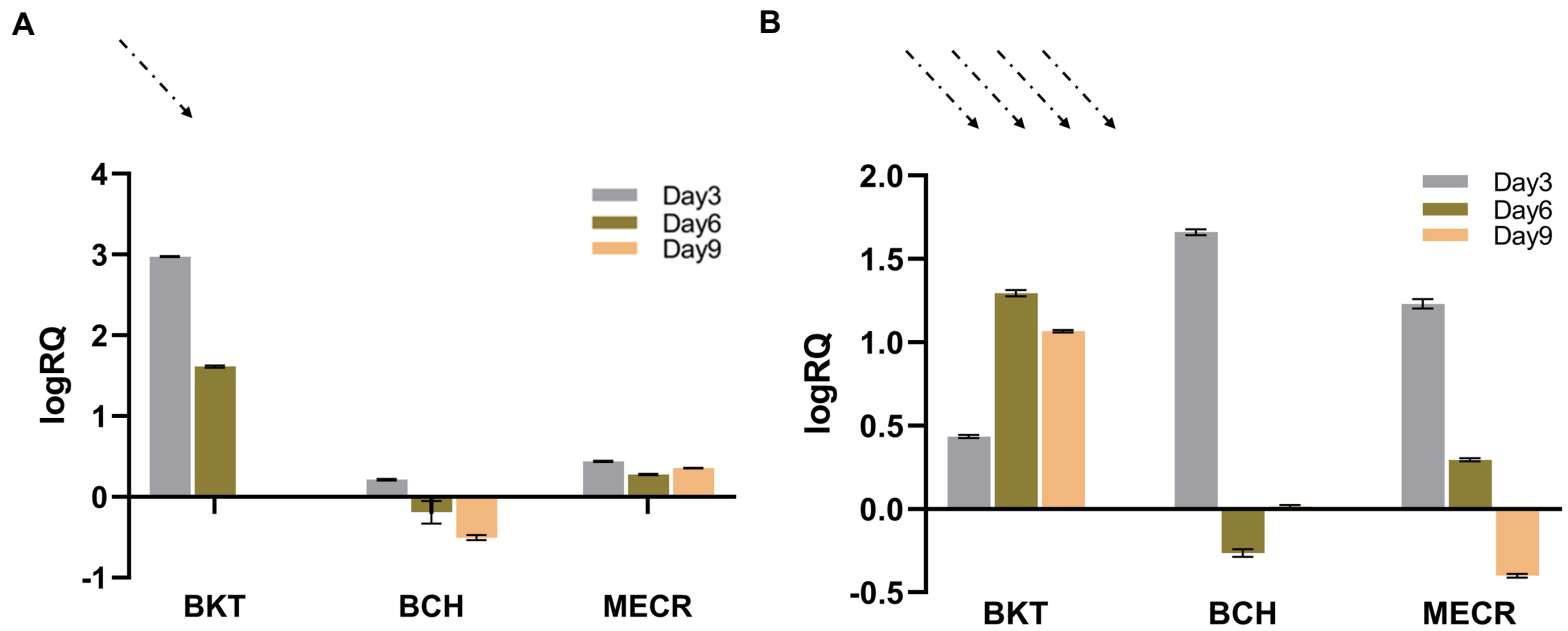
Results of real-time quantitative PCR. Dotted arrows indicate the intensity of light. **(A)** Results of 50 μmol·m^−2^·s^−1^ light intensity, logRQ>0 indicates that the gene expression is up regulated compared with that on day 0. logRQ<0 indicates that the gene expression is down regulated. **(B)** Results of 195 μmol·m^−2^·s^−1^ light intensity.

Real time fluorescent quantitative PCR was used to detect the transcription of foreign genes in T1 under 50 μmol·m^−2^·s^−1^ and 195 μmol·m^−2^·s^−1^ light intensity. The transcriptional level on the 3rd, 6th and 9th day were analyzed using the transcription level on the first day as the control. In general, *BKT* and *BCH* were successfully expressed in cells. Under normal light, the expression of *BKT* was higher than that of *BCH*, and the expression of both genes decreased with time. Under high light condition, the transcription level of each gene has been greatly improved. *BKT* gene was always at a high transcription level under high light intensity, and the *BCH* and *MECR* gene expression was significantly higher on the third day of high light treatment (Figure 2).

**Figure 3:**
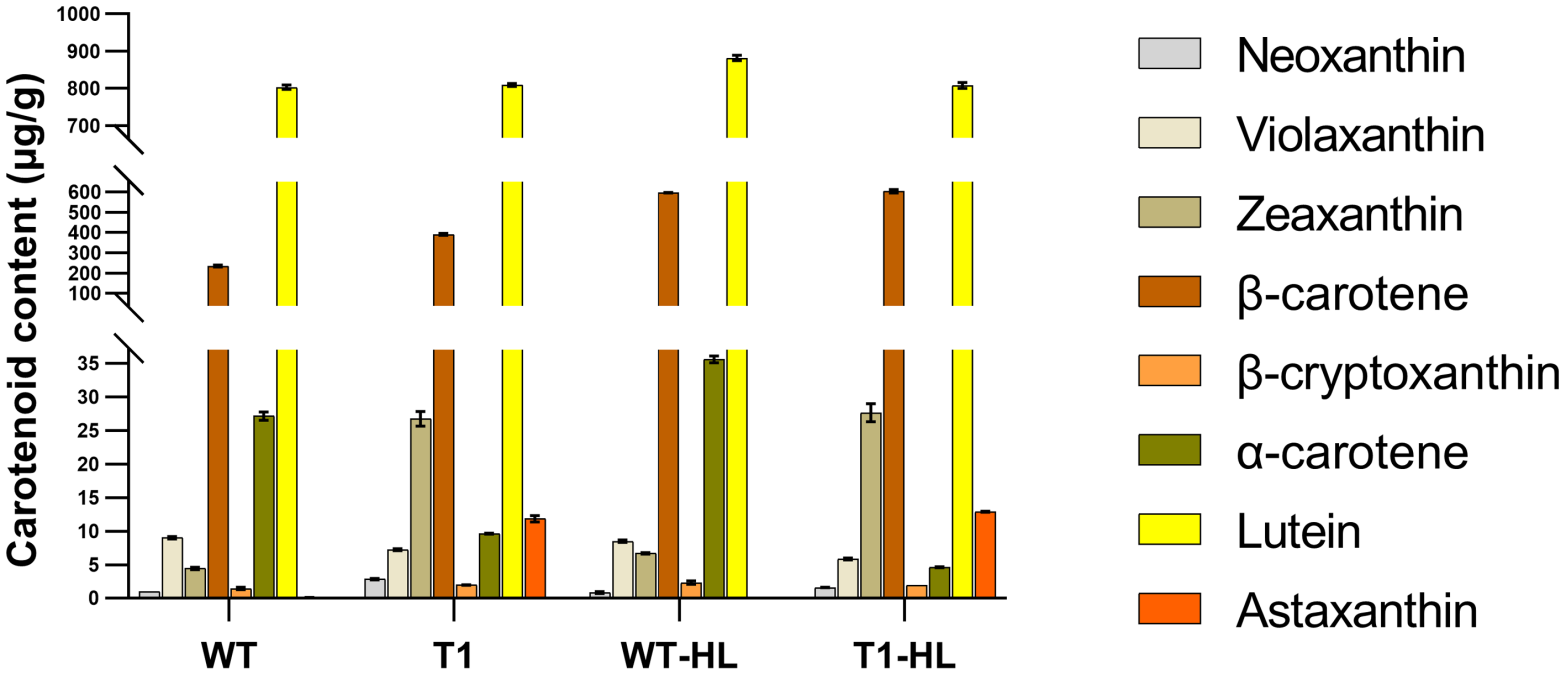
The content of carotenoids was determined by external standard HPLC. “HL” indicates the highlight condition. The vertical coordinate is segmented to make the appearance more appropriate.

Carotenoids in T1 and WT were measured by HPLC (Figure 3). Compared with WT, neoxanthin, zeaxanthin and astaxanthin in T1 are significantly increased under normal light and high light. Violaxanthin, α-carotene decreased significantly. β-cryptoxanthin increased significantly under normal light and decreased significantly under high light. Lutein did not change significantly under normal light, but decreased significantly under high light. β-carotene increased significantly under normal light, but did not change significantly under high light. In the carotenoid metabolic pathway, zeaxanthin is the common precursor of astaxanthin and violaxanthin. Therefore, we speculate that the expression of *BKT* and *BCH* leads to the redistribution of carbon flow, and more astaxanthin was synthesized, resulting in an increase of the synthesis of its precursors zeaxanthin. Similarly, the reduction of α-carotene may also be due to the redistribution of carbon flow. In general, high light led to the increase of carotenoid content, which indicates that there is a mechanism to avoid light damage by increasing carotenoid content in *C. reinhardtii*, which seems to be common in green algae. It is worth noting that astaxanthin content in WT can hardly be detected, which is consistent with previous studies. However, the significant increase of astaxanthin in T1 indicates that T1 can be used as a platform strain for later experiments.

### 3.2. Heterologous expression of genes related to fatty acid synthesis

**Figure 4:**
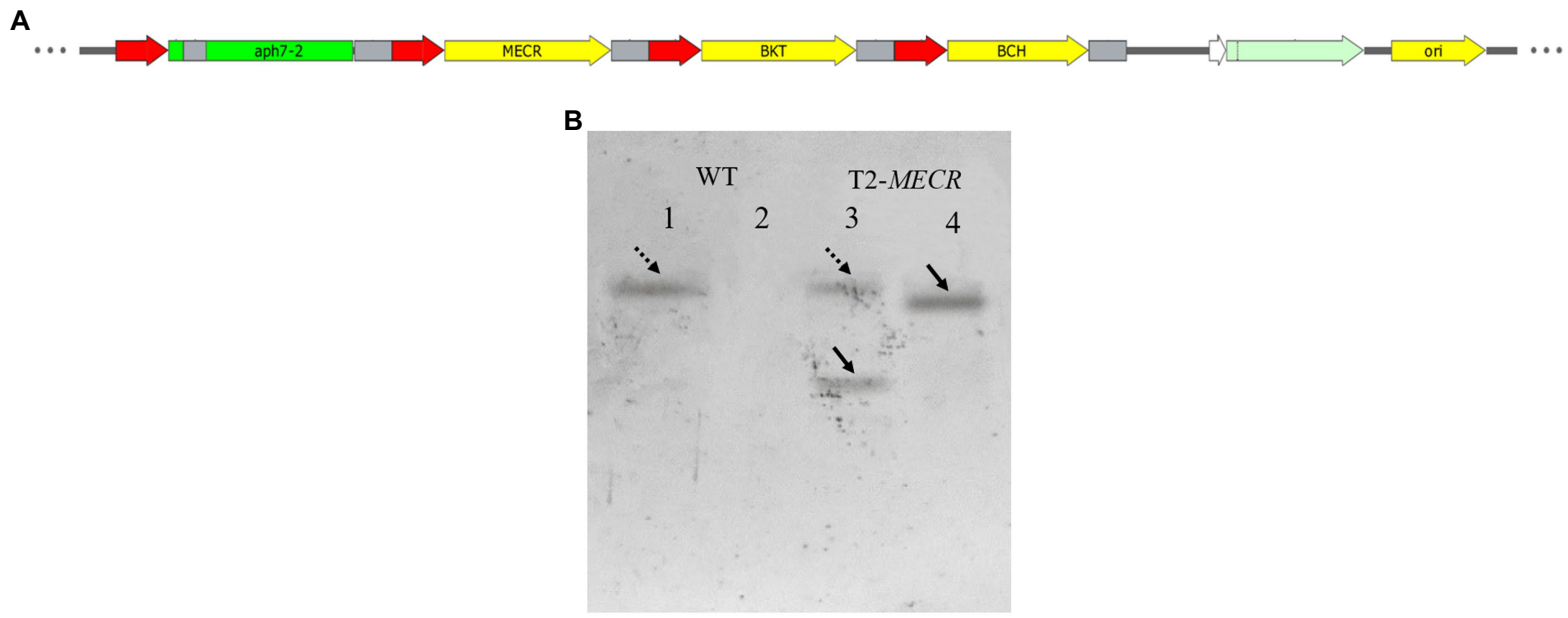
The structure of *MECR* gene expression vector and southern blotting results. **(A)** Use pHyg3-*BKT*-*BCH* as the skeleton, *MECR* was added. **(B)** Southern blotting results of *MECR* in T2. 1–2: WT. 3–4: T2. One and three were digested with *Xba*I/*Bam*HI. Two and Four were digested with *Nde*I/*Mlu*I. The dotted arrows indicate two bands in the same position.

**Figure 5:**
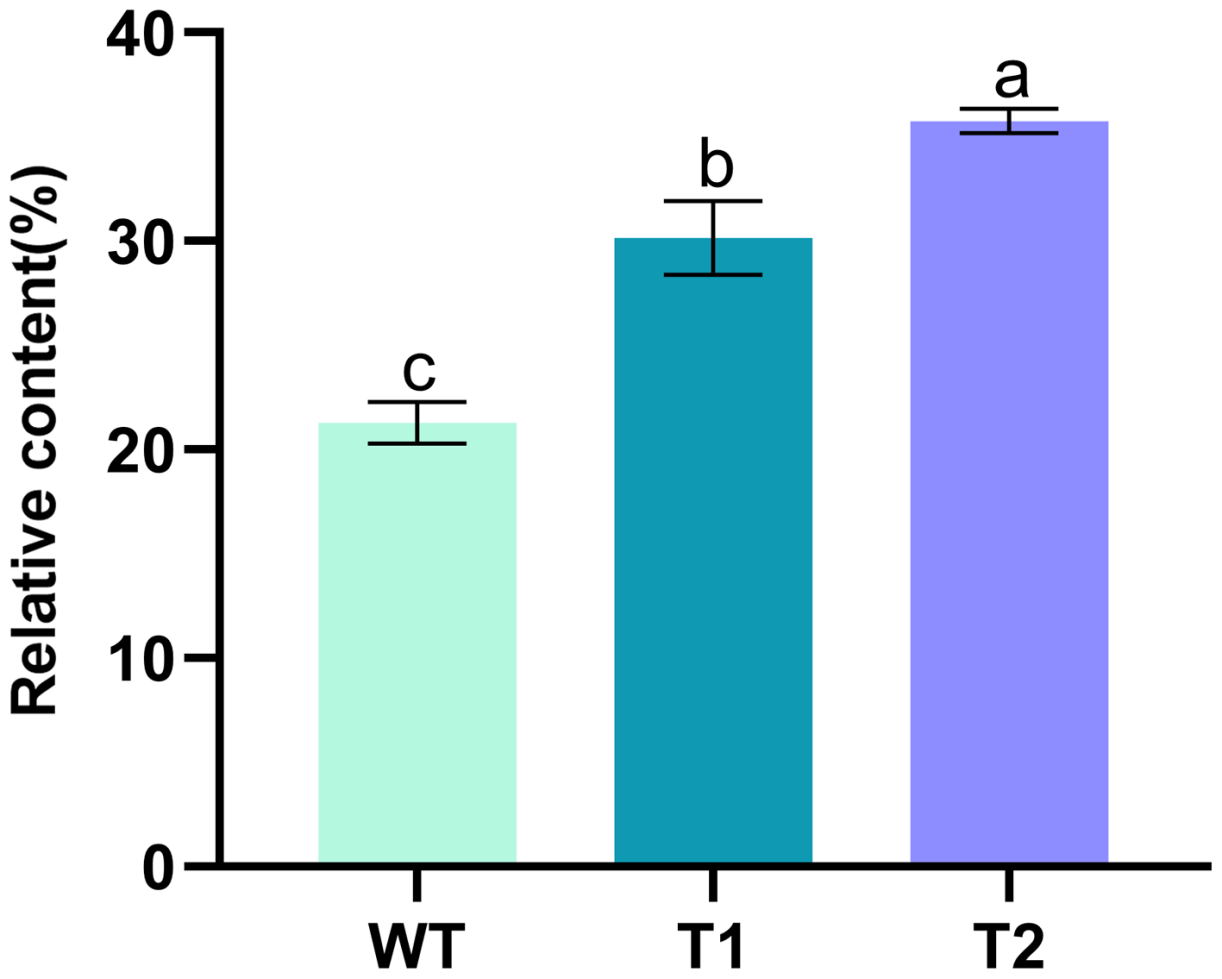
Mass percentage of total lipid content in WT and transformants. Lowercase letters indicate the significance of differences: the same letter indicates no significant difference, while different letters indicate significant differences (the same below).

On the basis of vector pHyg3-*BKT*-*BCH*, *MECR* gene was ligated with P_β2-Tub_ and T_rbcS2_ and then inserted into vector phyg3-*BKT*-*BCH* to get the vector pHyg3-*BKT-BCH-MECR* (Figure 4A). The final transformants are named as T2. Similarly, southern blotting was used to analyze the integration results of foreign genes. The genomic DNA of WT and T2 was digested by two groups of enzymes (*Xba*I/*Bam*HI and *Nde*I/*Mlu*I). One band appeared in WT digested with *Xba*I/*Bam*HI, and two bands appeared in T2 digested with the same enzyme, one of which was in the same position as WT. It was speculated that it might be a non-specific hybridization, and the other band was unique to T2, which should be a specific hybridization band of *MECR* gene. WT digested with *Nde*I/*Mlu*I showed no band, while T2 digested with *Nde*I/*Mlu*I showed a specific band. The above results demonstrate that *MECR* gene has been successfully integrated into T2 genome (Figure 4B). qPCR results also confirmed the successful expression of *MECR* gene (Figure 2). After obtaining appropriate transformants, the total lipid content in the transformants and WT was detected (Figure 5). The results showed that the total lipid in T1 which only transformed *BKT* and *BCH* was significantly higher than that in the WT, which indicated that the increase of astaxanthin promote the lipid accumulation. This result shows that the metabolic correlation between lipid and astaxanthin is mutual. In addition, on the basis of T1, the total lipid content of T2 strain transformed with *MECR* gene was significantly higher than that of T1. According to these results, *MECR* has been shown to promote total lipid.

**Figure 6:**
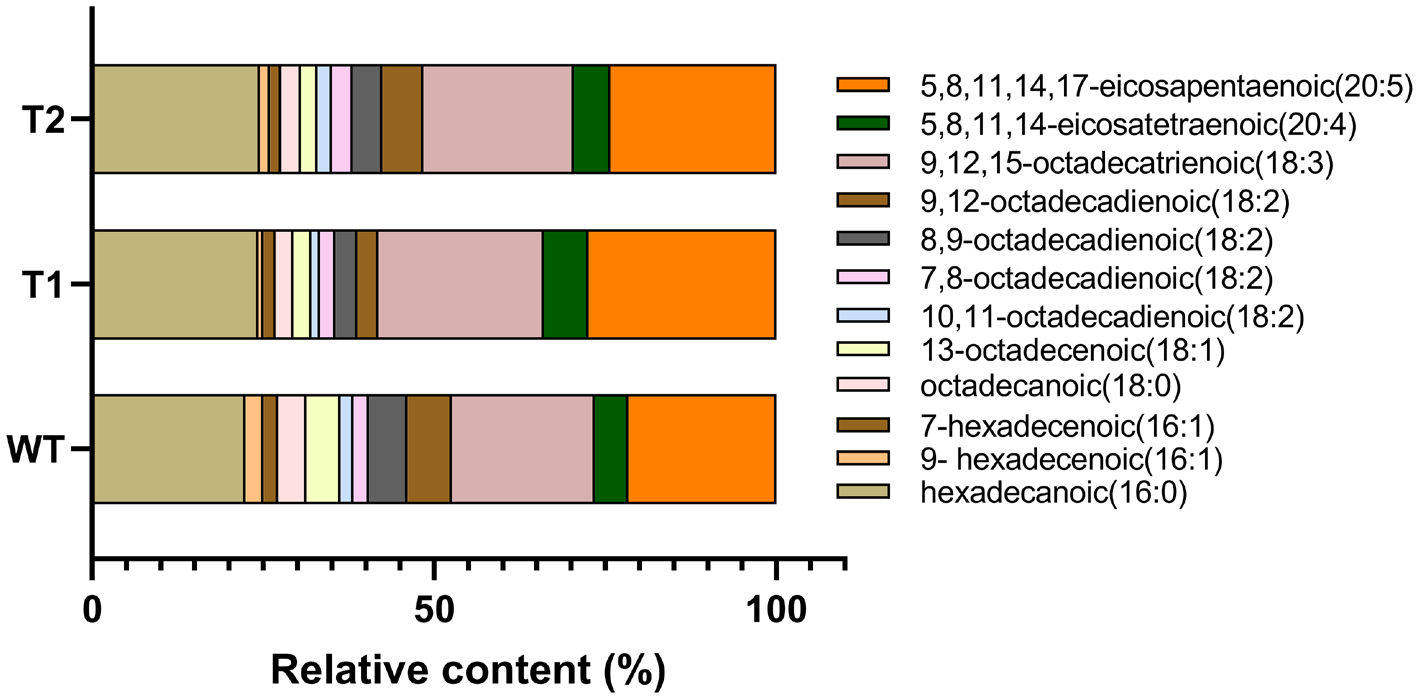
Statistical chart of relative content of various fatty acids based on GC–MS results. The original chromatogram can be found in Supplementary Material Figure 1.

**Figure 7:**
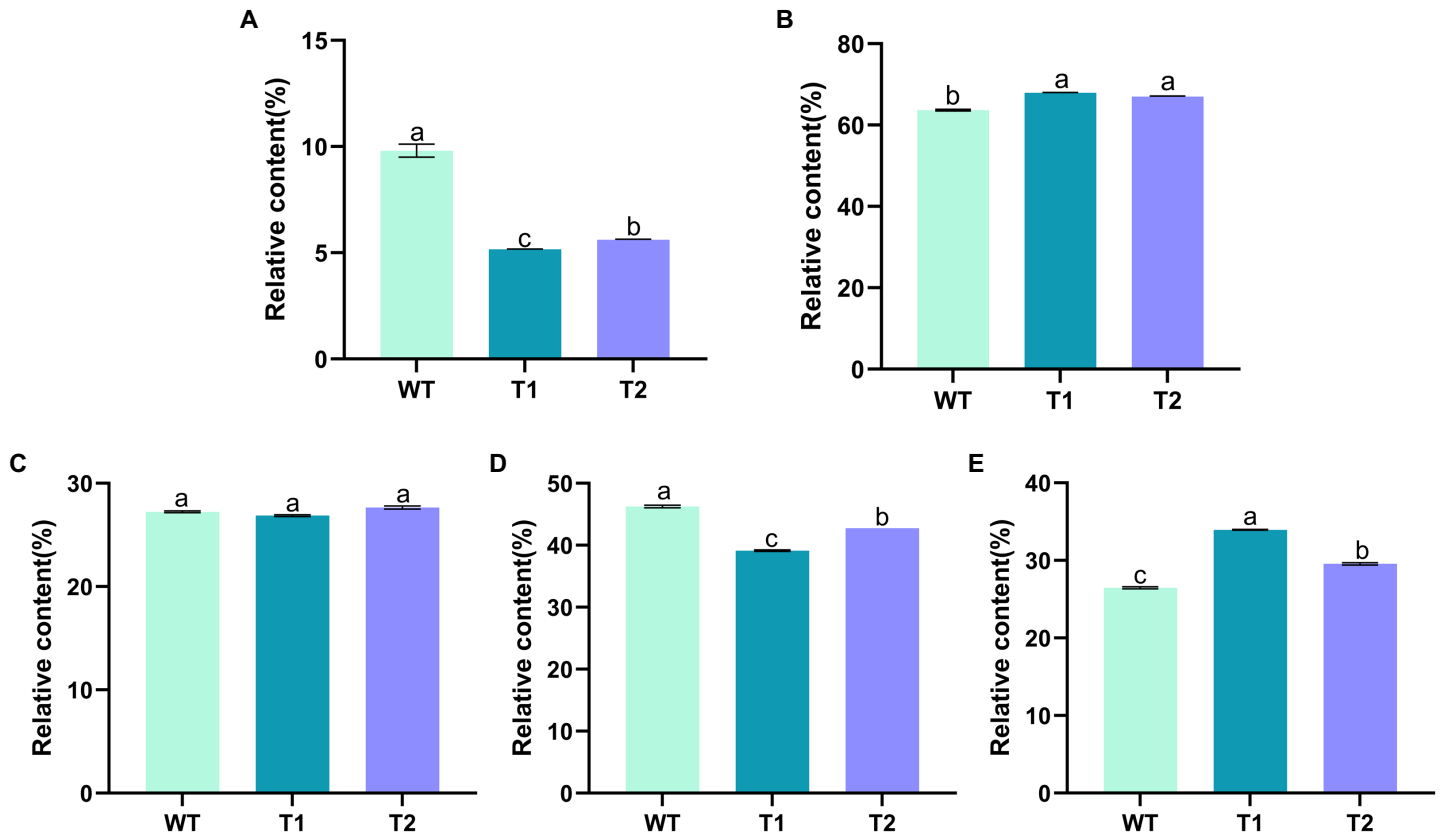
Relative content of fatty acids of different classifications. **(A)** Monounsaturated fatty acids. **(B)** Polyunsaturated fatty acids. **(C)** 16 C Fatty acids. **(D)** 18 C fatty acids. **(E)** 20 C fatty acids.

In order to determine the effect of different genes on the fatty acids in the transformants, GC–MS was used to measure the relative quantity of different fatty acids (Figure 6). The results showed that the fatty acids in WT were mainly hexadecanoic acid, octadecanoic acid and eicosanoic acid, including monounsaturated fatty acids and polyunsaturated fatty acids. The main fatty acids are hexadecanoic acid (Palmitic acid), 9,12,15-octadecatienoic acid (Linolenic acid) and 5,8,11,14,17-eicosapentaenoic acid (EPA), with the contents of 22.46, 20.95 and 21.61%, respectively. In addition, there were other unsaturated fatty acids with high nutritional value, such as 5,8,11,14-eicosatetraenoic (arachidonic acid). Overall, the composition of fatty acids of the transformants did not change compared with WT. The foreign gene did not change the type of fatty acids in *C. reinhardtii*, but the relative content of different fatty acids in the transformants changed. We compared the differences of fatty acid categories between transformants and WT. The results showed that monounsaturated fatty acids in T1 and T2 were significantly lower than those in wild type, but polyunsaturated fatty acids were significantly higher than those in wild type. Moreover, the content of monounsaturated fatty acids in T2 was higher than T1 (Figures 7A,B). In the comparison of fatty acids with different chain lengths, there was no significant difference in C16 between WT and transformants. The content of C18 in transformants was lower than that of WT, but T2 was higher than that of T1. The content of C20 in T1 and T2 was significantly higher than that in WT (Figures 7C,D,E). The above results indicate that *MECR* gene affect directly or indirectly the fatty acid elongation and desaturation metabolic pathway in *C. reinhardtii*. In addition, the change of fatty acid type in T1 further proves the influence of astaxanthin metabolism on lipid metabolism mentioned above.

### 3.3. Changes of astaxanthin content

**Figure 8:**
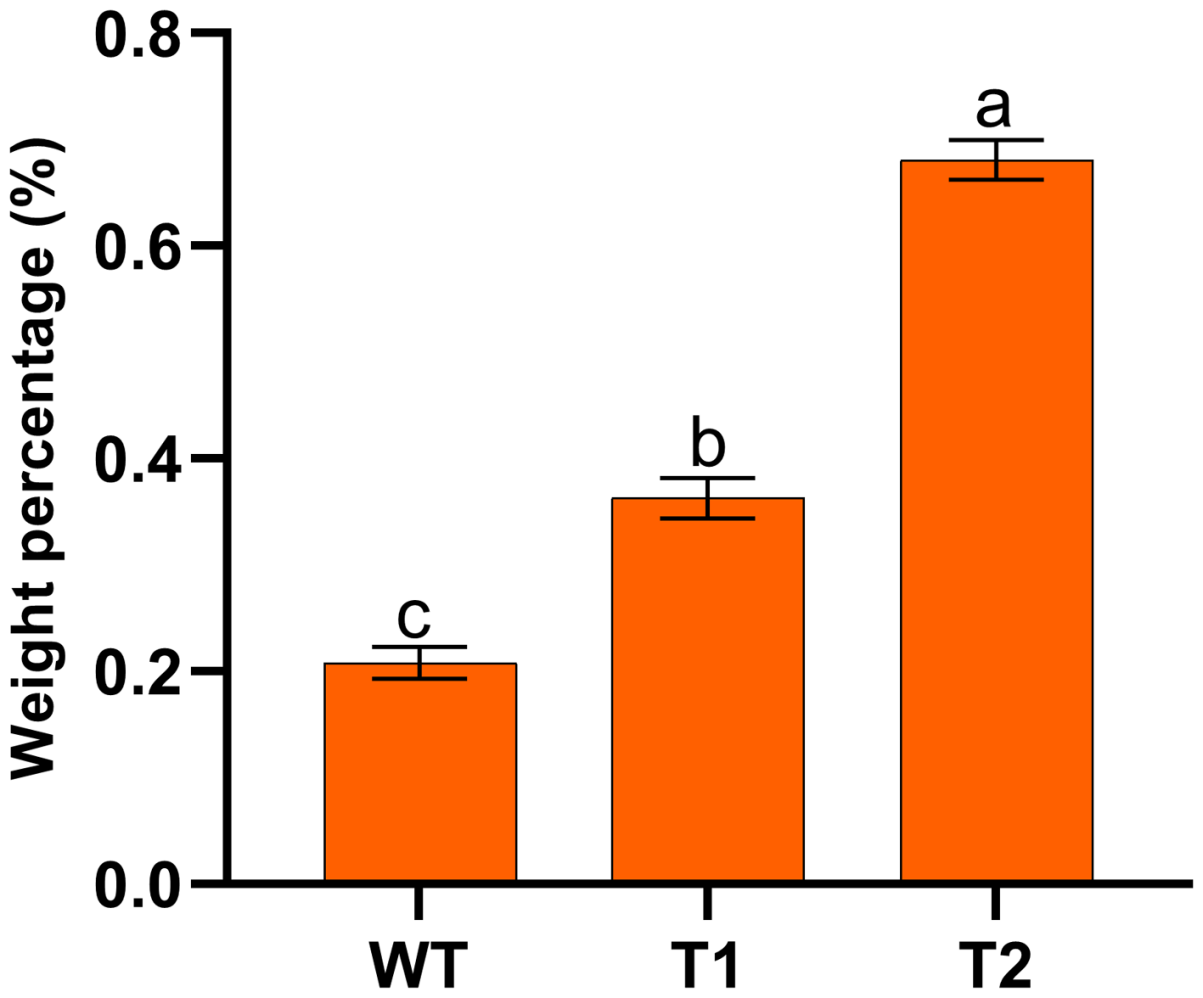
Mass percentage of astaxanthin in wild type and transformants.

Based on the above research, we have successfully expressed *H. pluvialis* genes that catalyze astaxanthin synthesis (*BKT*, *BCH*) and fatty acid elongation (*MECR*) in *C. reinhardtii*. Next, the effect of lipid increases and fatty acid changes on astaxanthin production were examined. The mass percentage of astaxanthin in transformants and WT was measured, as shown in Figure 8. We found that the astaxanthin content in T1 was significantly higher than that in WT, which was consistent with the liquid chromatography results in 3.1, with an increase of nearly 50%. In addition, the astaxanthin content in T2 was significantly higher than that in T1, which was 227.5%higher than that of wild type. These results indicate that the promotion of lipid production has indeed increased astaxanthin production. It is feasible to promote astaxanthin accumulation by transforming foreign gene *MECR*.

### 3.4. Growth of transformed algal strains

**Figure 9:**
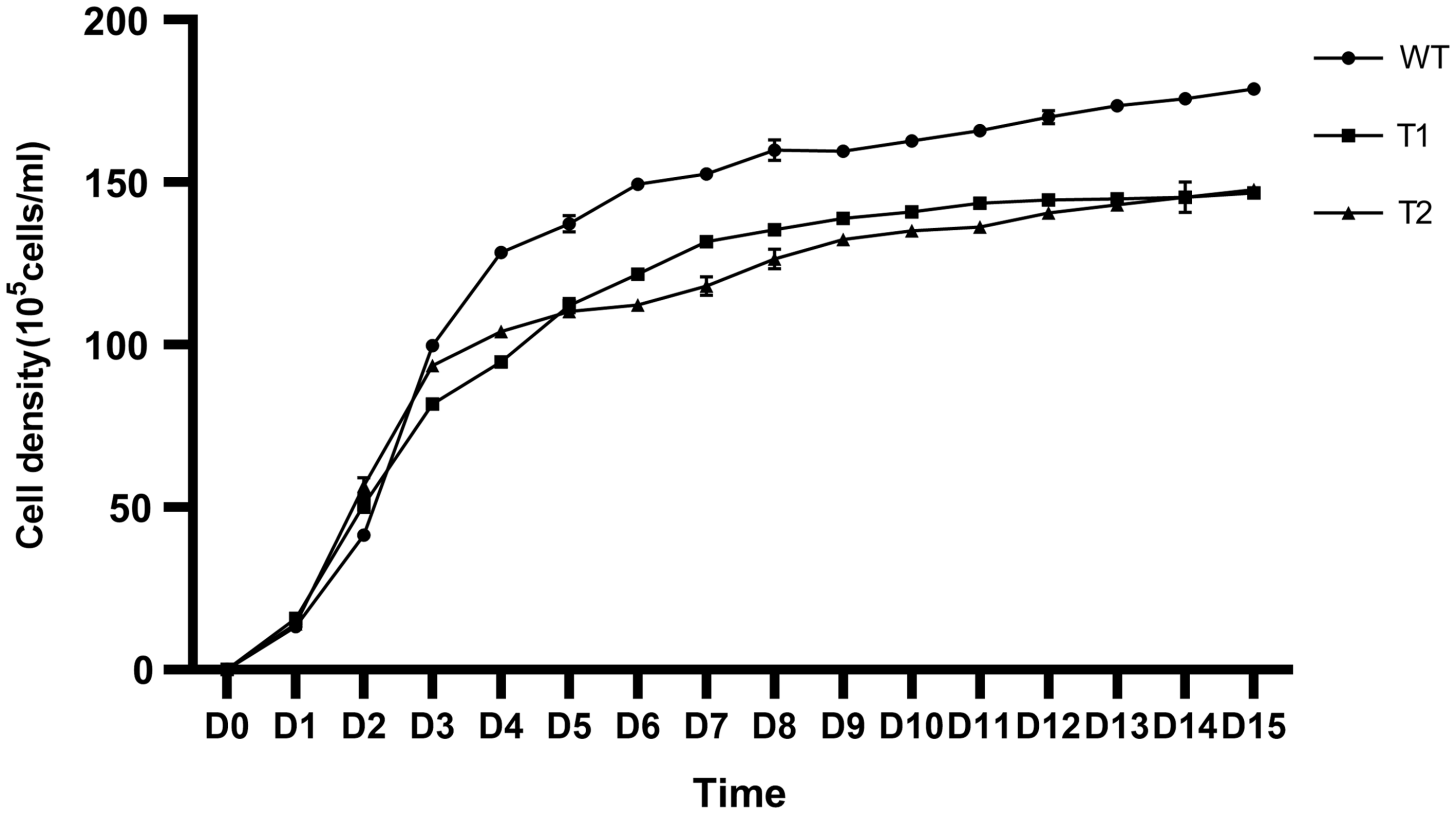
Growth curve of wild type and transformants from inoculation to the 15th day. The ordinate is the cell density.

**Figure 10:**
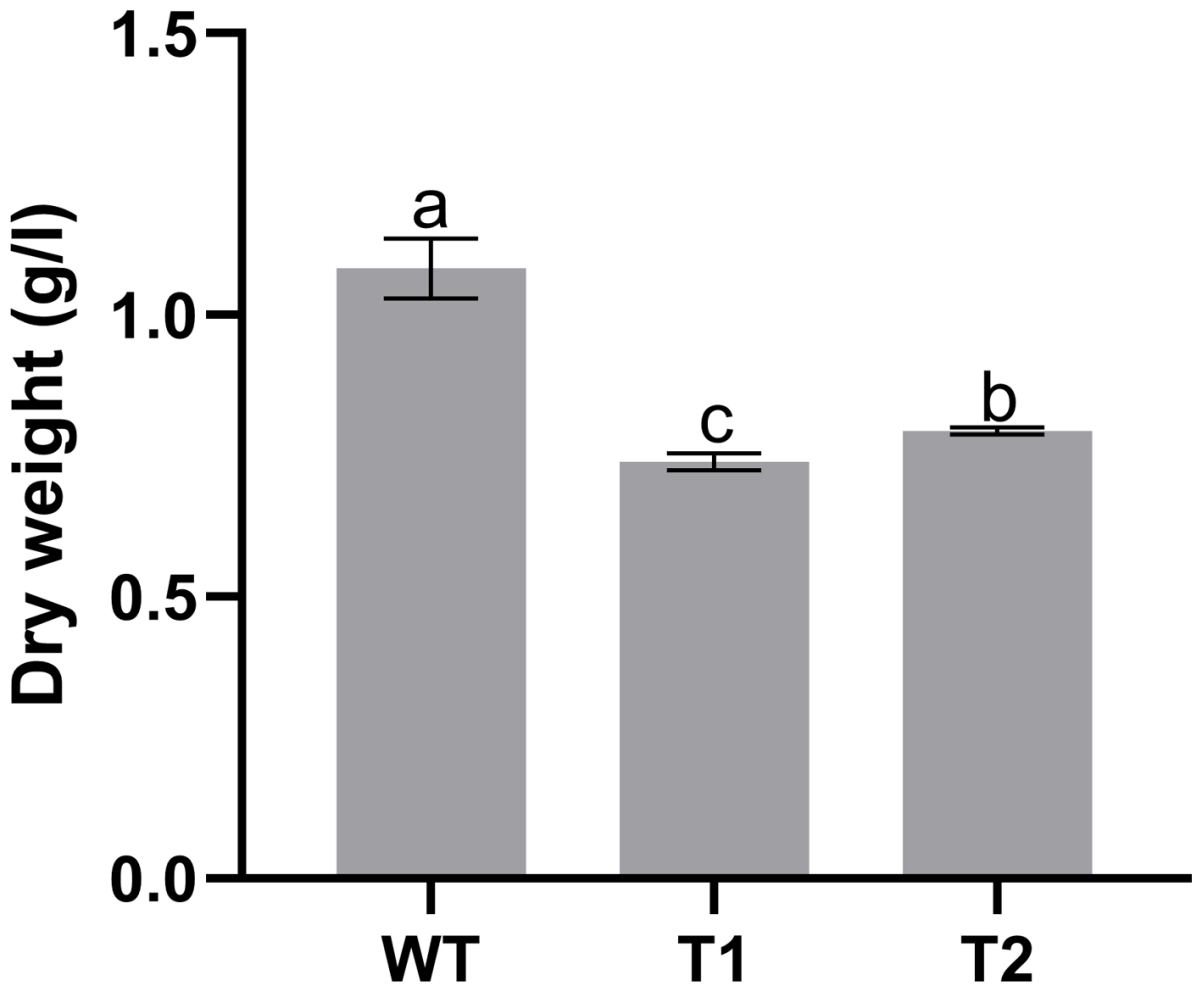
Dry weight of wild type and transformant.

In production, the growth of microalgae is a key indicator. We measured the growth curve and dry weight to characterize the growth of WT and transformant. The results showed that WT and transformants accumulated more than 50% biomass within 5 days after inoculation, then the growth rate slowed down and got into the platform period after 2 weeks of culture. However, we found that the biomass of the three transformants was significantly lower than that of WT, and there was no significant difference between different transformants (Figure 9). We measured the dry cell weight of algae after 15 days of culture, and the results were consistent with the growth curve. The dry weight of wild type was significantly higher than that of all transformants. And the dry weight of T2 is slightly higher than that of T1 (Figure 10).

## 4. Discussion

The huge demand for natural astaxanthin in the pharmaceutical, food and cosmetics industries has stimulated researchers to constantly seek more excellent astaxanthin producers. *H. pluvialis* is the most important source of natural astaxanthin, but the traditional cultivation method *of H. pluvialis* requires a lot of time and labor, resulting in high production costs. High prices make it is difficulty for more people to consume natural astaxanthin. To solve this problem, research of accumulation mechanism of astaxanthin in *H. pluvialis* and the cultivation of astaxanthin high-yield algae strains are necessary. In this study, *C. reinhardtii* was selected as the host to study the relationship between astaxanthin production and fatty acid metabolism. Up to now, many host organisms have been used to produce astaxanthin, including *E. coli*, *cyanobacteria*, etc. (23). As a model organism in modern microalgae research, *C. reinhardtii* has a clear genetic background and a large number of mature research tools and technologies have been applied in its molecular genetic research (24), which is the most promising cell factory (25). Therefore, *C. reinhardtii* can realize the transformation of foreign genes and provide carrier tools for studying the function of foreign genes. Benefiting from previous studies, nuclear transformation of *C. reinhardtii* has been constructed, and *Hyg* gene can be used as the main marker gene for nuclear transformation of *C. reinhardtii* (26, 27). For example, Hwangbo et al. overexpressed the FAB2 gene encoding stearyl ACP desaturase protein in *C. reinhardtii* through nuclear transformation, and found that the oleic acid (18:1n9) content of the mutant algal strain obtained was about 2.4 times higher than that of the control algal strain (33). Tan and Lee (28) expressed the fatty acyl ACP thioesterase gene of *Dunaliella terminolecta* in *C. reinhardtii*. The results showed that the total lipid content of the transgenic algae strain increased by about 56%, and the normal growth of the transgenic algae strain was not affected. The experimental results of this study also proved the reliability and convenience of the glass bead conversion method. Then, because this kind of transgenic is non-targeted, the insertion of foreign gene may cause unknown changes in the host gene, which is not conducive to the analysis results. In this study, among the transformants with different phenotypes, the representative transformants were selected for analysis by comparing the growth status of different transformants and the expression level of foreign genes. Heterogeneous production of astaxanthin in *C. reinhardtii* by expressing key genes in *H. pluvialis* astaxanthin synthesis pathway (such as *BKT* and *BCH*) has been proved (5, 29, 30). However, no significant astaxanthin production was obtained in previous studies. It is speculated that this may be due to the feedback regulation of carotenoid content by cells, inappropriate localization of foreign gene expression products in cells, and the use of inappropriate promoters.

**Figure 11:**
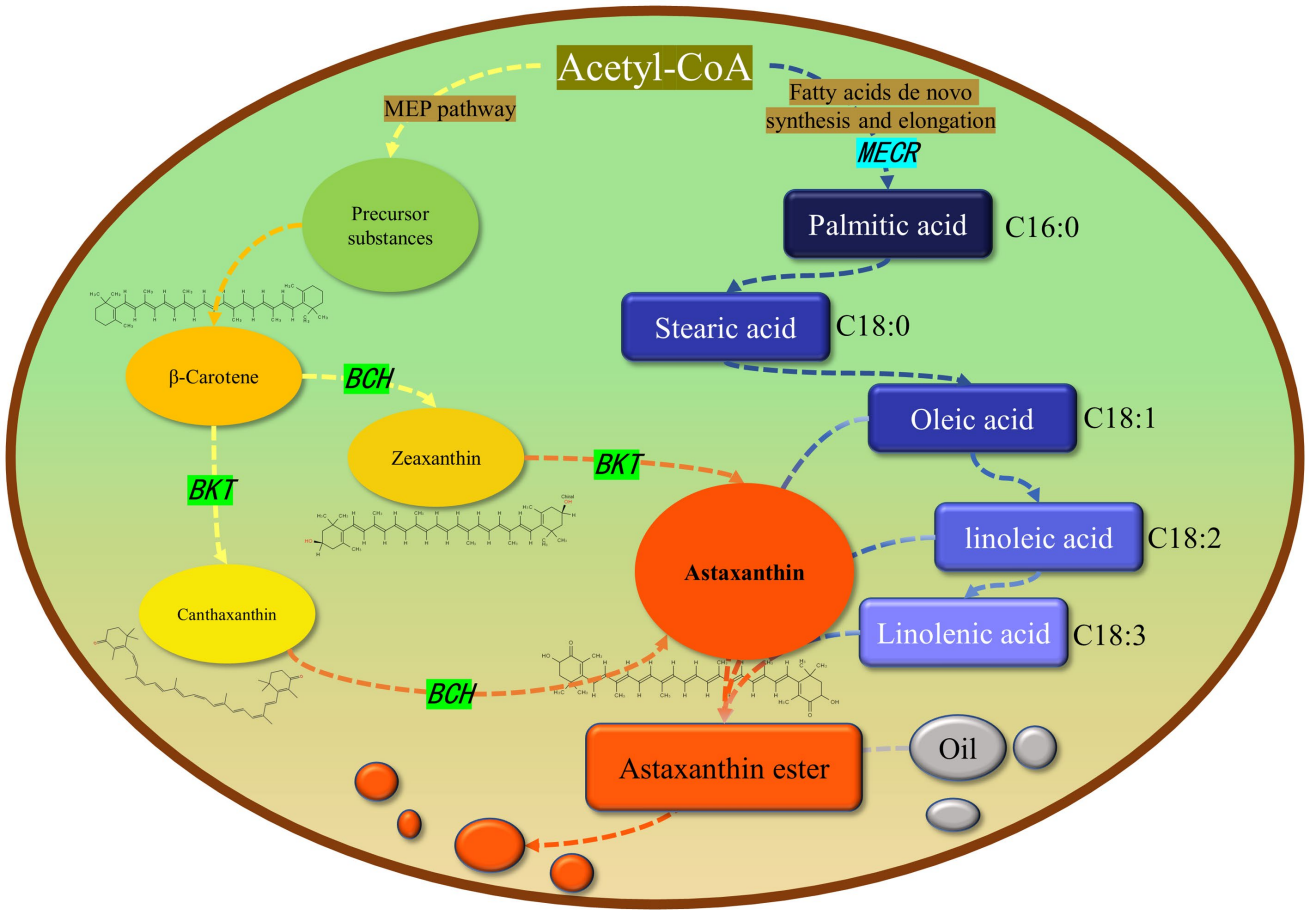
Relationship between astaxanthin and lipid metabolism in *Haematococcus pluvialis.* Most astaxanthin is esterified with fatty acids to form astaxanthin ester, which is then dissolved in oil droplets in cells and diffused in cells. Green and light blue indicate the genes we operate on and their location in the metabolic pathway. The arrow represents a logical relationship. We only show the content related to our research.

In this study, *MECR* gene related to fatty acid elongation in mitochondria was co-transformed with astaxanthin synthesis genes (*BKT* and *BCH*) into *C. reinhardtii* to increase astaxanthin production by promoting the production of lipid. The yield of astaxanthin was significantly increased in *C. reinhardtii*. Figure 11 summarized our experimental ideas. According to previous studies, the final form of astaxanthin in *H. pluvialis* is the astaxanthin esters with a variety of fatty acids, in which monoesters account for the majority, diesters and free astaxanthin account for only a few. Monounsaturated and polyunsaturated fatty acids of the C18 family (C18:1, C18:2 and C18:3) are the main lipids (14). According to previous studies, *MECR* gene is responsible for the elongation of fatty acids from C4 to C16 in mitochondria (31, 32). In this study, the content of C16 in *C. reinhardtii* was not significantly increased after heterologous expression of *MECR*. On the contrary, the relative content of C20 increased significantly. This may be due to the influence of intracellular regulation mechanism, so that the increased C16 was used to synthesis longer chain fatty acids. In addition, the gene was named as MECR because of its high similarity (50%) to the MECR amino acid sequence of *Chlorella sorokiniana.* In fact, 2-enoyl thioester reductase (ETR) is the main domain in it. ETR domain is found in many species and catalyzes the NADPH-dependent conversion of trans-2-enoyl acyl carrier protein/coenzyme A (ACP/CoA) to acyl-(ACP/CoA) in fatty acid synthesis. The specific function and the active site in *H. pluvialis* need to be further studied, however there is no doubt that it can increase the proportion of long-chain fatty acids. Initially, we hoped to promote astaxanthin production by increasing the content of long-chain fatty acids (more than 12 carbon atoms) without changing the total amount of lipid. However, GC–MS analysis showed that the genes transformation changed the proportion of different long-chain fatty acids, the unsaturation of fatty acids and the total amount of lipid. Therefore, the increase of astaxanthin content in T2 may be related to many reasons about fatty acids. On the other hand, we found that the foreign gene that we operated changed the relative content of different fatty acids in *C. reinhardtii*, which may be an inspiration for using microalgae to produce fatty acids with high nutritional value.

Through the transcriptional level detection and analysis of *MECR*, *BKT* and *BCH* in transgenic algal strain T2, we found that three foreign genes were successfully transcribed. In addition, the transcriptional level of foreign genes were up-regulated after high light treatment. It can be speculated that the up-regulation of the transcription level of the above three genes may be related to the native metabolic pathway of carotenoid synthesis and fatty acid in *C. reinhardtii*. Since we did not use inducible promoters to regulate the expression of foreign genes, this shows that the difference in gene expression under high light is affected by the regulatory mechanism of *C. reinhardtii* itself.

In addition, the growth performance of the transformants we obtained is weaker than that of the wild type. This result may be caused by the incompatibility between the foreign gene and the host. From the material point of view, the newly established astaxanthin metabolic pathway and fatty acid metabolic pathway redistribute the original carbon flow, resulting in the reduction of biomass. From the perspective of energy, the synthesis of astaxanthin requires ATP, which consumes the energy originally used for biomass accumulation and leads to the reduction of biomass. In order to obtain engineering algae strains that can be used to produce astaxanthin, this defect needs to be solved. It may be possible to use promoters that are more compatible with the host, select more critical genes or combine transgenic and mutagenic screening to cultivate new mutants.

## 5. Conclusion

In this research, *C. reinhardtii* was used to produce astaxanthin by expressing *BKT* and *BCH* genes related to astaxanthin synthesis. On this basis, *MECR* gene related to the formation of long-chain fatty acids were transformed. Our results confirmed the increase of astaxanthin production in the transformed algal strain. It has important significance to understand the relationship between astaxanthin metabolism and lipid metabolism in microalgae. In addition, it provides a good reference for molecular biology breeding of *H. pluvialis* for increasing astaxanthin yield.

## Data availability statement

The datasets presented in this study can be found in online repositories. The names of the repository/repositories and accession number(s) can be found at: https://www.ncbi.nlm.nih.gov/genbank/, MN561260, https://www.ncbi.nlm.nih.gov/genbank/, MN561261.

## Author contributions

J-pS is responsible for the integration of whole research and the writing of manuscript, and carried out some experimental operations. X-hW is responsible for the transformation of *MECR* gene and the acquisition of algal strain T2. X-mC is responsible for transforming *BKT* and *BCH* genes into *Chlamydomonas reinhardtii* CC849 and obtains T1. W-hZ and L-XQ are responsible for the preservation and cultivation of algae strains. X-nZ is responsible for guiding the whole study and revising the manuscript. All authors contributed to the article and approved the submitted version.

## Funding

This research is Supported by the National Natural Science Foundation of China (32273112).

## Conflict of interest

The authors declare that the research was conducted in the absence of any commercial or financial relationships that could be construed as a potential conflict of interest.

## Publisher’s note

All claims expressed in this article are solely those of the authors and do not necessarily represent those of their affiliated organizations, or those of the publisher, the editors and the reviewers. Any product that may be evaluated in this article, or claim that may be made by its manufacturer, is not guaranteed or endorsed by the publisher.

## Supplementary material

The Supplementary material for this article can be found online at: https://www.frontiersin.org/articles/10.3389/fnut.2023.1130065/full#supplementary-material
