# Supplementary material for: Expression of fatty acid related gene promotes astaxanthin heterologous production in Chlamydomonas reinhardtii
Source: Front Nutr. 2023 Mar 20;10:1130065. doi: 10.3389/fnut.2023.1130065 (PMC10067919; doi:10.3389/fnut.2023.1130065)
Supplement: Supplementary file 1 [file Data_Sheet_1.docx]

Supplementary Material

Promote Heterologous Production of Astaxanthin in Chlamydomonas reinhardtii by Combining Fatty Acid Metabolism

Jin-peng Sun ^1^, Xue-hong Wei ^2^, Xiao-mei Cong ^2^, Wen-hua Zhang ^3^, Le-Xin Qiu ^3^, Xiao-nan Zang **

*** Correspondence:** ZANG Xiao-nan: xnzang@ouc.edu.cn

# Supplementary Figures and Tables

## Supplementary Figures


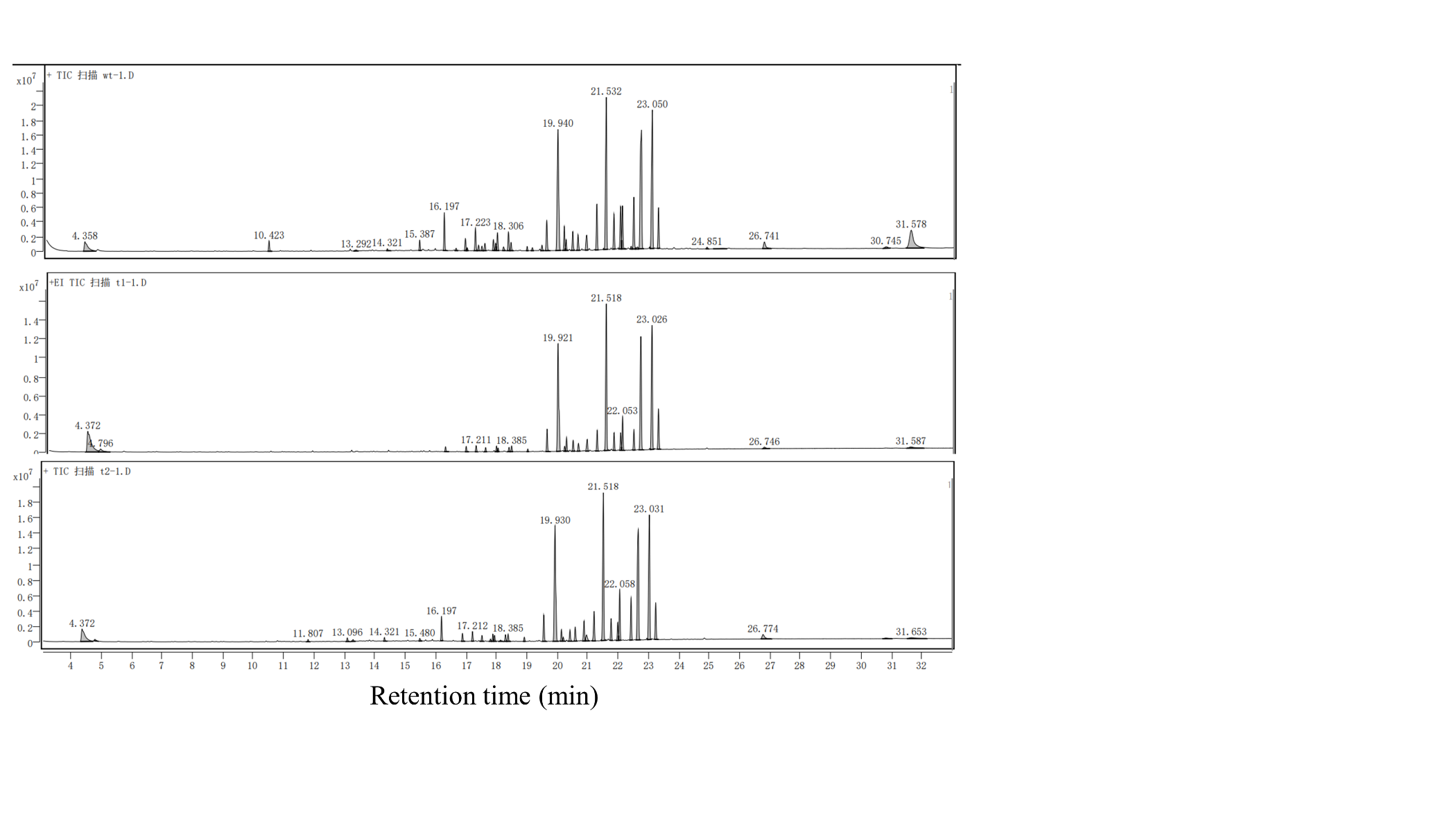


**Supplementary Figure 1.** Gas chromatograms of WT, T1 and T2. The number on the peak indicates the retention time. The peak area is integrated to calculate the relative content of each fatty acid.

## Supplementary Tables

**Table S1**. The primers and digestion sites used to clone the target gene, and the underlined mark indicates the sequence of the digestion sites.

| Primer name | Sequence |
| --- | --- |
| *BCH*- *Bam*HI | GGATCCATGCTGTCGAAGCTGCAGTCAATCA |
| *BCH*- *Sac*I | GAGCTCCTAGTGGTGGTGGTGGTGGTGCCGCTTGGACCAGTCCAGTTC |
| *BKT*-*Bam*HI | GGATCCATGCACGTCGCATCGGCACTAATG |
| *BKT*-*Sac*I | GAGCTCCTAGTGGTGGTGGTGGTGGTGTGCCAAGGCAGGCACCAG |
| MECR-*Xba*I | TCTAGAATGCAGGCCTGCCGGCACATCGCG |
| MECR-*Bam*HI | GGATCCTCAGTGGTGGTGGTGGTGGTGTGAGGACAGCATGTC |
| P_β2-Tub_ -*Hin*dIII | AAGCTTGAATTCGATATCAAGCTTCTTTCT |
| P_β2-Tub_-*Sal*I | GTCGACGTTTGCGGGTTGTGACTGA |
| T_rbcS2_-*Sac*I | GAGCTCTAAGGATCCCCGCTCCGTGTA |
| T_rbcS2_-*Sac*I | GAGCTCAAGCTTCCATGGGATGACGGG |

Table S2. Endonucleases used to digest genomic DNA in southern blotting.

| Gene | Endonucleases |
| --- | --- |
| *BKT* | *Sac*I/*Hin*dIII; *Sal*I/*Bam*HI |
| *BCH* | *Mlu*I/*Bgl*II; *Xho*I/*Nde*I |
| *MECR* | *Xba*I/*Bam*HI; *Nde*I/*Mlu*I |

Table S3. Primers used to make southern blotting probes.

| Primer name | Sequence |
| --- | --- |
| *BKT*-probe-F | CGGCACTAATGGTCGAGCAGAA |
| *BKT*-probe-R | AAGCTGGGCTGTGGCTTCG |
| *BCH*-probe-F | CTGCAGTCAATCAGCGTCAAGG |
| *BCH*-probe-R | GCAGCCTGGTATGACAGCTG |
| *MECR*-probe-F | AGCCAAGCTGGGTCCAACGCC |
| *MECR*-probe-R | CTCCAGTAGAGGGTGCGGCTAG |

Table S4. Primers for real-time quantitative PCR.

| Primer name | Sequence |
| --- | --- |
| *BKT*-F | GCACTAATGGTCGAGCAGAAAG |
| *BKT*-R | TGCGGTCCAGGTGCCAATGA |
| *BCH*-F | CTGCAGTCAATCAGCGTCAAGG |
| *BCH*-R | GCAGCCTGGTATGACAGCTG |
| *MECR*-F | GCCAACGCTGCCAGGGACAC |
| *MECR*-R | TGGAAGGCTGAGGCACGCACC |
| *RCK*-F | CTTCTCGCCCATGACCAC |
| *RCK*-R | CCCACCAGGTTGTTCTTCAG |
